# Malignancy-Induced Hypercalcemia—Diagnostic Challenges

**DOI:** 10.3389/fped.2017.00233

**Published:** 2017-11-13

**Authors:** Claire Hoyoux, Jacques Lombet, Corina Ramona Nicolescu

**Affiliations:** ^1^Department of Pediatric Hematology and Oncology, Centre Hospitalier Regional de la Citadelle, University of Liège, Liège, Belgium; ^2^Department of Pediatric Nephrology, Centre Hospitalier Regional de la Citadelle, University of Liège, Liège, Belgium; ^3^Department of Pediatric Endocrinology, Centre Hospitalier Regional de la Citadelle, University of Liège, Liège, Belgium

**Keywords:** hypercalcemia, malignancy, parathyroid hormone, bone lymphoma, parathyroid hormone-related peptide

## Abstract

Hypercalcemia in children is a rare metabolic finding. The clinical picture is usually non-specific, and the etiology includes several entities (metabolic, nutritional, drug-induced, inflammatory, cancer-associated, or genetic) depending on the age at presentation, but severe hypercalcemia is associated mainly with malignancy in childhood and sepsis in neonates. Severe parathyroid hormone (PTH)-suppressed hypercalcemia is challenging and requires multidisciplinary diagnostic and therapeutic approaches to (i) confirm or rule out a malignant cause, (ii) treat it and its potentially dangerous complications. We report a case of severe and complicated PTH-independent hypercalcemia in a symptomatic 3-year-old boy. His age, severity of hypercalcemia and its complicated course, and the first imaging reports were suggestive of malignancy. The first bone and kidney biopsies and bone marrow aspiration were normal. The definitive diagnosis was a malignant-induced hypercalcemia, and we needed 4 weeks to assess other differential diagnoses and to confirm, on histopathological and immunochemical base, the malignant origin of hypercalcemia. Using this case as an illustrative example, we suggest a diagnostic approach that underlines the importance of repeated histology if the clinical suspicion is malignancy-induced hypercalcemia. Effective treatment is required acutely to restore calcium levels and to avoid complications.

## Introduction

Severe hypercalcemia in children is a medical emergency and often manifests as non-specific symptoms such as nausea, vomiting, weight loss, and anorexia.

Most patients with hypercalcemia are found to have endocrine disorders (hyperparathyroidism and calcium-sensor anomalies), vitamin D intoxication (real or linked to genetic disease), as well as inflammatory, malignant, or genetic entities. Although common in adults, malignancy-induced hypercalcemia is rarely encountered at the time of the diagnosis in childhood (<1% of pediatric cancers) (1) and its main biological feature is suppressed secretion of parathyroid hormone (PTH). Different tumor types can induce hypercalcemia, with acute leukemia, lymphoma, and solid tumors being the most prevalent (2).

Several mechanisms are thought to be responsible for the pathophysiology of cancer-associated hypercalcemia. The most discussed mechanism is the production of parathyroid hormone-related peptide (PTHrP) with bone and kidney actions along with increasing calcium levels. There is no relationship between serum levels of PTHrP and hypercalcemia because it demonstrates endocrine (some PTH-like) effects and paracrine (direct PTHrP osteoclastic activity) effects (3).

Regardless of etiology, managing hypercalcemia involves identification of the underlying cause and reducing plasma levels of calcium to avoid/minimize end-organ consequences. An acute therapeutic approach is required if symptoms, complications, or very high calcium levels are present. The principle of this intervention relies on enhancing physiological excretion of calcium and paying attention to the child’s hydration status. First-line treatment includes rehydration and loop diuretics, but bisphosphonates and corticosteroids could be required as second-line treatment. Dialysis is an emergency intervention.

We present a case of complicated malignancy-induced hypercalcemia and underline the etiological approach and diagnostic difficulties, mostly if the first biopsies are normal.

## Case Report

A 3-year-old boy was admitted to the emergency department with a 2-day history of abdominal pain and vomiting in the context of isolated constipation. He did not have other symptoms (fever, bone pain, polyuria, weight loss, or lethargy). He was otherwise well and was not taking any medication. His medical history was unremarkable as was that of his family.

Upon hospital admission, he was unwell with normal vital signs, except high blood pressure (126/80 mmHg). There were no remarkable findings upon physical examination except for a distended abdomen with diffuse tenderness, but no rebound or organomegaly.

Laboratory evaluation upon hospital admission was notable for a potassium level of 2.2 mmol/L, pH of 7.44, bicarbonate level of 30.3 mmol/L, and urea level of 50 mg/dL. Blood count was normal. Abdomen radiography showed multiple lytic lesions throughout the visualized thoracic (ribs) and basin skeleton (iliac bones).

The child was admitted for additional laboratory analyses and imaging studies. The most important results and their evolution are shown in Table 1. Biological results were notable for an increased total (4.57 mmol/L) and ionized (2.33 mmol/L) levels of calcium in the context of persistent hypokalemia and metabolic alkalosis as well as severely reduced levels of vitamin D, 1,25-dihydroxycholecalciferol [1,25 (OH)_2_D] and PTH (5 ng/mL). The full blood count was normal. A shortened QT interval (but not dysrhythmia) was noted on several electrocardiograms. An abdominal ultrasound indicated bilateral symmetric kidney enlargement with mild nephrocalcinosis. Computed tomography (CT) revealed well-defined radiolucent bone lesions of varying size without a sclerotic rim at the ribs, pelvic bones, proximal femoral epiphysis, or metaphysis. Other abnormal findings (adenopathy, visceromegaly, solid mass, and lung lesions) were not described.

**Table 1:** From admission to diagnosis—biochemical, radiological, and histological evolution.

Variables and reference range	ED laboratories	Laboratories and radiology on 1st week	Laboratories on 2nd week	Laboratories and radiology on 3rd week	Laboratories on 4th week/diagnosis
Blood count	Normal, no peripheral blasts	Normal, no peripheral blasts	Normal, no peripheral blasts	Normal, no peripheral blasts	Normal, no peripheral blasts
Acid–base balance	pH 7.44	pH 7.45			
Total serum calcium (2.1–2.6 mmol/L)	4.57	4.16–4.68	2	3	
Ionized calcium (1.2–1.3 mmol/L)	2.33	2.53	1.76	2.11	
Parathyroid hormone (15–65 ng/mL)	5	8			
Parathyroid hormone-related peptide (<20 pg/mL)	<20		<20		
Vitamin D (≥30 ng/mL)	8	14			
1,25(OH)_2_D (20–80 pg/mL)	6.3	7.5			
Tumor markers (CRP, LDH, hCG, αFP, and NSE)	Negative		Negative		Negative
Abdominal X-ray	Multiple osteolytic lesions (iliac bones and ribs)				
Abdominal US	Bilateral homogenous nephromegaly (longitudinal diameter 9 cm)				
Thorax and abdominal CT		Multiple radiolucent bone lesions Normal thorax and lungs			
Bone scintigraphy		Normal			
18F-PET/scan		Normal			
Skeleton X-ray				Multiple lytic lesions Femoral fractures T7 vertebral compression	
Biopsies	Bone marrow aspiration, kidney, and iliac lesions biopsy—normal				Knee lesions and bone marrow aspiration—lymphoblast infiltration

*CRP, C-reactive protein; LDH, lactic dehydrogenase; hCG, human chorionic gonadotropin; αFP, alpha-fetoprotein; NSE, neuron-specific enolase; 18F-PET/scan, ^18^F-fluorodeoxyglucose positron emission tomography/computed tomography; CT, computed tomography; US, ultrasound*.

These biological (PTH-suppressed hypercalcemia) and imaging (disseminated lytic bone lesions and symmetrical nephromegaly) findings were highly suggestive of malignancy. However, the first biopsies (posterior iliac crest and kidney biopsies, and bone marrow aspiration) failed to demonstrate abnormal cells.

A repeated blood cell count and panel of tumor markers (C-reactive protein, lactate dehydrogenase, uric acid, human chorionic gonadotropin, alpha-fetoprotein, and neuron-specific enolase) were also normal. Bone scintigraphy (^99m^Tc) demonstrated no uptake in osteolytic lesions and ^18^F-fluorodeoxyglucose positron emission tomography/computed tomography did not identify hyper-metabolic activity.

Other causes of PTH-suppressed hypercalcemia were investigated: hypophosphatasia, lymphangiomatosis, and granulomatous diseases. None of the features in the child’s history, physical, biological, or imaging work-up were suggestive of a specific cause.

**Figure 1:**
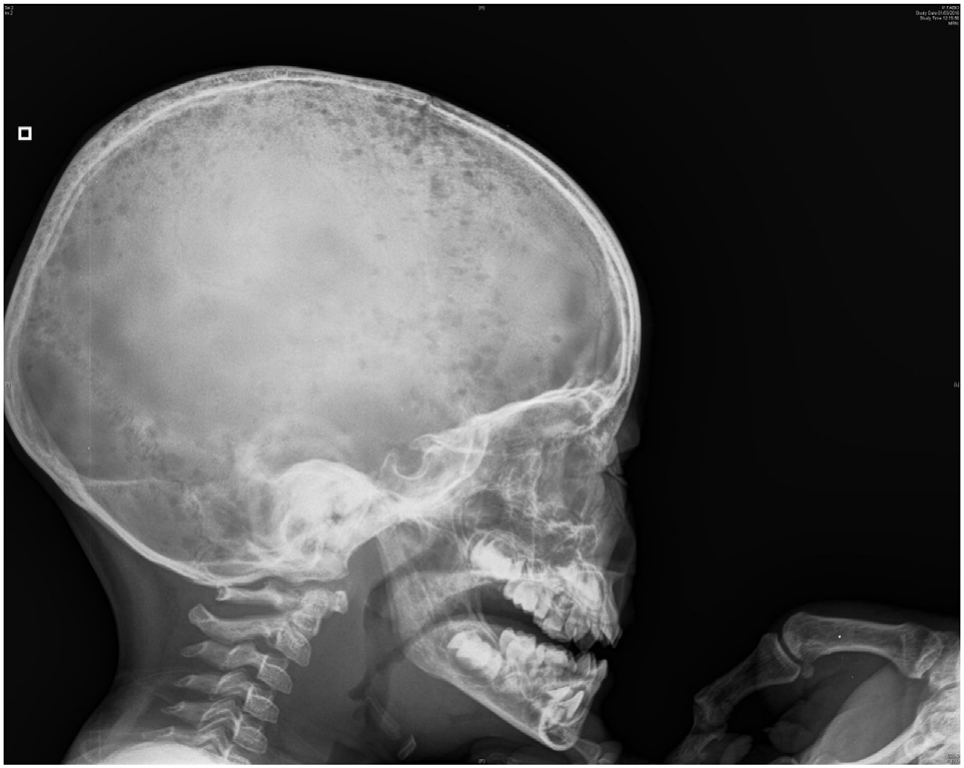
Skull radiograph—multiple lytic areas.

His clinical evolution was marked by two complications. In the second week of hospitalization, he developed severe abdominal pain with increases in levels of pancreatic enzymes (hypercalcemia-induced pancreatitis). One week later, he refused to walk, with progressively increasing bilateral knee pain. Full-skeleton radiography showed supplementary lytic lesions involving the skull (Figure 1) and knees, with bilateral distal femoral fractures and vertebral compression at the T7 level. At this time, a surgical bone biopsy (knee lesions) and a second bone marrow aspiration were undertaken and showed lymphoblast infiltration. Bone marrow histology revealed a blast population expressing CD45dim, CD34+, HLA DR+, CD10+, cCD22+, cCD97a+, CD24+, TdT+, but not CD19, sCD22, or CD20. Markers of T cells and NK cells were also negative. These findings were suggestive of primary bone pre-B cell lymphoblastic lymphoma with bone involvement of the bone marrow (stage IV) and confirmed the diagnosis of malignancy-induced hypercalcemia. The blood count remained normal with no blasts on peripheral smear at the time of the histological diagnosis.

Primary bone pre-B lymphoblastic lymphoma is a rare form of malignancy and bone marrow involvement is even rarer. Bone lesions are, along with skin or subcutaneous infiltrations, the most frequent clinical presentations (4). The vertebrae, pelvis, ribs, and the ends of long bones are the preferred destinations of metastases because of their high content of red marrow.

**Figure 2:**
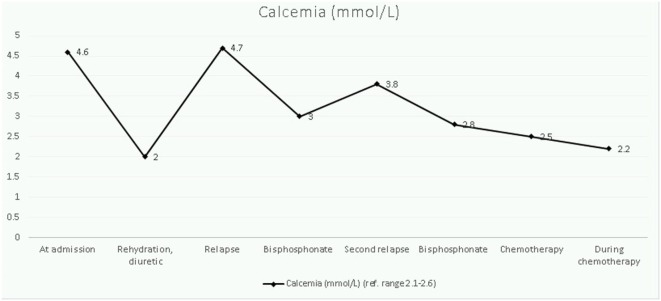
Longitudinal evolution of calcemia.

The therapeutic approach of this severe hypercalcemia started 48 h after admission and was oriented to reduce calcium levels progressively, normalize potassium homeostasis, and to control cardiac complications. Intravenous hydration (0.9% saline, 3 L/m^2^/24 h) with potassium chloride (3 mmol/kg/day) was started on day 2 of hospitalization and had no effect on calcium levels. After 2 days, the kalemia normalized and a diuretic furosemide, 1 mg/kg/dose was added. Serum levels of calcium declined gradually, but a first rebound was noticed rapidly. Bisphosphonate therapy (one dose of pamidronate at 1 mg/kg, iv) was started on day 7 and resulted in partial correction of calcium levels. However, on day 15, he redeveloped hypercalcemia and a 3-day pamidronate cure (0.5 mg/kg/day) was given, without subsequent hypocalcemia being noted. At this time, malignancy had not been excluded, so corticosteroids were not administered. Once the diagnosis had been established, hypercalcemia was treated with induction chemotherapy (Figure 2).

At the time of writing this report, the child is under chemotherapy and evolution (clinical, biological, and imaging) is favorable. Bilateral nephromegaly is present and the blood pressure is normal, without antihypertensive medication.

## Discussion

Severe hypercalcemia reflects a serious disruption of calcium homeostasis, and its end-organ complications (cardiac, neurological, and renal) represent a metabolic emergency necessitating an immediate diagnosis-oriented exploration and correction of electrolyte abnormalities.

The first step of the diagnostic algorithm is a biochemical evaluation including plasma and urine levels of calcium (with urine calcium/creatinine ratio), PTH, vitamin D, and phosphate. A standard biological check-up (hematologic, renal, hepatic, electrolytes, and acid–base balance) is also required (5), along with adrenal and thyroid measurements. The PTH level is particularly helpful for the etiological approach (5).

The second step is imaging work-up, including abdominal ultrasound and whole-skeleton radiography. CT, bone scintigraphy (^99m^Tc), and PET/CT evaluation could be necessary to offer consistent morphological and metabolic assessments.

A third step, particularly if suppressed PTH levels are present, requires tissue biopsy with histological and immunochemical analyses.

In our case (PTH-suppressed hypercalcemia with multiple osteolytic bone lesions), the diagnosis of malignant hypercalcemia was highly suspected. The normal biochemical profile in the absence of malignant cells on three tissue (bone marrow, bone, and kidney) samples obliged us to make a differential diagnosis with other entities associated with a suppressed level of PTH, hypercalcemia, and lytic bone lesions.

Hypophosphatasia is caused by an inactivated mutation of *ALPL* that results in low alkaline phosphatase activity as well as defective bone and tooth mineralization (6). Hypophosphatasia was excluded on the basis of normal specific biological markers (pyridoxal 5′-phosphate, urine, and serum levels of phosphoethanolamine).

One possible explanation for the moderate hypophosphatasemia in our case was suppressed phosphatase activity in the skeleton in the setting of long-lasting disseminated lytic lesions with progressive destruction and resorption.

Lymphangiomatosis is characterized by diffuse or multicentric proliferation of dilated lymphatic vessels and has a variable clinical presentation, affecting the bone, liver, spleen, mediastinum, lungs, and soft tissues. Osteolysis is the most common finding with the axial skeleton (skull, spine, and ribs) being involved preferentially (7), and hypercalcemia could be secondary to bone resorption, insufficient mineralization in the bone matrix, as well as enhanced intestinal or renal absorption.

In granulomatous diseases, activation of T-cells and macrophages leads to excessive endogenous synthesis of 1,25(OH)_2_D and excessive bone resorption and gut absorption of calcium (8).

In a child with severe PTH-suppressed hypercalcemia, the suspicion of malignancy should be kept in mind, even if the biological and imaging features render this possibility unlikely.

Repeated biopsies are, ultimately, the required tool to make histological and immunochemical diagnoses.

Two concomitant mechanisms have been discussed in the pathogenesis of malignancy-induced hypercalcemia: (a) osteolysis due to proliferation and bone-tissue invasion by tumor cells and (b) osteoclastic bone resorption induced by tumor humoral factors (PTHrP and cytokines) (1). Adult studies have demonstrated increased mRNA levels for PTHrP in cultured leukemic cells, as well as increased plasma levels in patients with T-cell leukemia and B-cell lymphoma (9) However, there is no direct correlation between PTHrP production in malignant cells, its bone effects, and serum levels of calcium. In our case, the PTHrP levels were normal and this finding could not exclude PTHrP involvement in the pathogenesis of hypercalcemia. The PTHrP molecule is degraded rapidly extracellularly and can act in a paracrine way to increase osteoclastic activity (10). A lower prevalence of increased PTHrP levels in hematopoietic malignancies compared with those in solid tumors in adults has been documented (10).

In our case, the interpretation of normal bone scintigraphy and PET was difficult. Rajarubendra and colleagues reported that in adults bone scintigraphy assesses osteoblastic processes rather than tumor proliferation and, consequently, false-negative results can occur (11). Furthermore, primary osteolytic lesions with limited reactive osteoblastic reactions and certain well-differentiated tumors can go undetected by ^18^F-FDG because of poor accumulation of ^18^ F-FDG (12).

A particular feature of humoral hypercalcemia of malignancy is the associated metabolic alkalosis and hypokalemia. The alkalosis might be due to buffers (calcium carbonate and phosphate) released from bone with metastatic lesions (13).

In 1977, Aldinger and coworkers (14) showed that malignant-induced hypercalcemia is frequently associated with hypokalemia.

In 2010, a review addressing the role of the calcium-sensing receptor (CaSR) in the physiology and pathophysiology of the kidneys (13), described the effects of hypercalcemia on monovalent and divalent cation transport and renine release. During hypercalcemia, activation of the basolateral CaSR inhibits the renal outer medullary potassium (ROMK) channels responsible for the recycling of potassium ions into the lumen of the thick ascending limb.

The more severe the hypercalcemia, the greater is the inhibition of ROMK and Bartter syndrome-like effects.

From a therapeutic perspective, the combination of hypokalemia and metabolic alkalosis in the setting of hypercalcemia is challenging. The first step of hypercalcemia treatment should be limited to hydration and supplementation with potassium chloride and should not include furosemide.

## Conclusion

Severe symptomatic hypercalcemia could be the first manifestation of a bone-localized malignancy, but its accurate and definitive diagnosis remains challenging.

While waiting for histological results, etiological discussion of the non-malignant causes of PTH-suppressed hypercalcemia could be necessary and reassuring.

## Ethics Statement

This case report was approved by the Ethics Committee of the Centre Hospitalier Regional de la Citadelle (University of Liege, Liege, Belgium). The parents of the child gave written informed consent in accordance with the Declaration of Helsinki.

## Author Contributions

CH, JL, and CN contributed equally to the conception and design of this work. The acquisition of data, the drafting of the manuscript, the final elaboration, and critical revision were made by CN.

## Conflict of Interest Statement

The authors declare that the work was conducted in the absence of any commercial or financial relationships that could be construed as a potential conflict of interest.
